# Epithelial cells as alternative human biomatrices for comet assay

**DOI:** 10.3389/fgene.2014.00386

**Published:** 2014-11-28

**Authors:** Emilio Rojas, Yolanda Lorenzo, Kristiane Haug, Bjørn Nicolaissen, Mahara Valverde

**Affiliations:** ^1^Depto. Medicina Genómica y Toxicología Ambiental, Instituto de Investigaciones Biomédicas, Universidad Nacional Autónoma de MéxicoMéxico, México; ^2^Department of Ophthalmology, Center for Eye Research, Oslo University Hospital, Ullevål, University of OsloOslo, Norway

**Keywords:** comet assay, human epithelial cells, comet sampling

## Abstract

The comet assay is a valuable experimental tool aimed at mapping DNA damage in human cells *in vivo* for environmental and occupational monitoring, as well as for therapeutic purposes, such as storage prior to transplant, during tissue engineering, and in experimental *ex vivo* assays. Furthermore, due to its great versatility, the comet assay allows to explore the use of alternative cell types to assess DNA damage, such as epithelial cells. Epithelial cells, as specialized components of many organs, have the potential to serve as biomatrices that can be used to evaluate genotoxicity and may also serve as early effect biomarkers. Furthermore, 80% of solid cancers are of epithelial origin, which points to the importance of studying DNA damage in these tissues. Indeed, studies including comet assay in epithelial cells have either clear clinical applications (lens and corneal epithelial cells) or examine genotoxicity within human biomonitoring and *in vitro* studies. We here review improvements in determining DNA damage using the comet assay by employing lens, corneal, tear duct, buccal, and nasal epithelial cells. For some of these tissues invasive sampling procedures are needed. Desquamated epithelial cells must be obtained and dissociated prior to examination using the comet assay, and such procedures may induce varying amounts of DNA damage. Buccal epithelial cells require lysis enriched with proteinase K to obtain free nucleosomes. Over a 30 year period, the comet assay in epithelial cells has been little employed, however its use indicates that it could be an extraordinary tool not only for risk assessment, but also for diagnosis, prognosis of treatments and diseases.

## Introduction

In this special issue, we review the use of the comet assay to map DNA damage in different human cells since the inception of this field nearly 30 years ago by Ostling and Johanson (1984). The aim of the present review is to summarize data published in the meantime that address the use of this tool in evaluating DNA damage in cells other than blood mononuclear cells. An increasing number of studies are being published in this area, particularly with respect to life style, environmental, and occupational exposure risk evaluations, as well as therapeutic interventions, promoting the use of the comet assay as an additional suitable human biomarker.

At the International Workshop on Genotoxicity Test Procedures (IWGTP), which was held in Washington, DC in 1999, an expert panel met to develop guidelines for the use of the comet assay in genetic toxicology. The expert panel reached a consensus that the optimal version of the assay for identifying genotoxic activity was the alkaline (pH > 13) version of the assay that was developed by Singh et al. (1988). This version of the comet assay is capable of detecting DNA single-strand breaks (SSB), alkali labile sites (ALS), DNA-DNA/DNA-protein cross-linking, and SSB associated with incomplete excision repair sites. The advantages of the comet assay relative to other genotoxicity tests include its sensitivity in detecting low levels of DNA damage; the requirement of a small number of cells per sample; and its flexibility, ease of application, and short duration. The expert panel identified the minimal experimental and methodological standards required to ensure that the results of comet studies would be accepted as valid by knowledgeable scientists and regulatory agencies (Tice et al., 2000).

It is important to note that only one study addressing human monitoring was published between 1988 and 1993, which was a review article authored by McKelvey-Martin et al. (1993). Subsequently, periodical publications regarding lifestyle and human exposure studies have greatly increased, the majority of which were included in the review articles published in 1999 (Rojas et al., 1999) and 2009 (Valverde and Rojas, 2009).

More recently, the launch of the ComNet project during the International Comet Assay Workshop (ICAW) meeting in Kusadasi, Turkey proposed the aim of establishing the comet assay as a reliable and trusted biomarker assay (Collins et al., 2012). The first ComNet project publication focused on the use of the comet assay as a tool for human monitoring, assuming some difficulty in validating previously published data. The most important disadvantages of the studies were the small number of subjects and the discrepancies in the methodological aspects applied in different laboratories around the world using blood cells (Collins et al., 2014).

To avoid the previously identified variations resulting from the use of blood cells, the few human biomonitoring comet assay studies using epithelial cells allow us to review the protocols and observe the methodological conditions that optimized their use and enhanced their application.

The present review aims to construct a set of widely acceptable guidelines that may help to eliminate much of the experimental variation that has generated the large heterogeneity of comet assay data and frustrates attempts to compare and combine studies in different laboratories using the comet assay in different types of epithelial cells (specifically, lens, corneal, tear duct, buccal, and nasal epithelial cells).

Epithelial cells, as specialized components of many organs, have the potential to serve as biomatrices that can be used to evaluate genotoxicity and may also serve as early effect biomarkers; furthermore, 80% of solid cancers are of epithelial origin. Epithelial cells are characterized by common structural features (specifically, their arrangement into cohesive sheets), but have diverse functions that are made possible by many specialized adaptations. Many of the physical properties of epithelial cells are dependent upon their attachment to one another, which is mediated by several types of cell junctions. The specialized functions of epithelial cells are mediated through both structural modifications of their surfaces and internal modifications, which adapt cells to fulfill their specific roles, ranging from absorption to secretion to serving as a barrier.

The surface epithelia and the epithelia of many simple glands belong to the continuously renewing cell population. The rate of cell turnover is characteristic of the specific epithelium; for example, small intestinal cells are renewed every 4–6 days in humans. The stratified squamous epithelium of the skin is replaced approximately once every 28 days (Ross and Pawlina, 2006), nasal epithelial cells are replaced approximately once every 30 days and buccal epithelial cells are renewed approximately once every 10–14 days. However, other epithelial cells, particularly those in more complex glands or tissues, may survive for a long time (Kruze, 1994; Ross and Pawlina, 2006; Chiego, 2014).

All of these specialized modifications of the epithelia necessitate various modifications to the comet assay procedure to obtain a single cell suspension, a limiting step in performing the assay using these types of cells.

According to International Program of Chemical Safety (IPCS) guidelines (Albertini et al., 2000), the optimal sample collection timing for any cell population is during long-term chronic exposure when the induction and repair of DNA damage is presumed to be maintained at steady-state equilibrium; such timing maximizes the likelihood that an agent can be identified as DNA damaging. For the sampling of cells after an acute exposure or after termination of chronic exposure to a genotoxic agent, the optimal collection time for detecting induced DNA damage is most likely within a few hours of exposure termination; this window of sampling can affirm that the extent of DNA damage in a population of cells decreases as the amount of time between exposure termination and sampling increases. In addition, the repair of DNA damage through DNA repair processes and the loss of heavily damaged cells through apoptosis, necrosis, or cell turn over are also dependent upon the agent of exposure. An additional advantage of the comet assay for human biomonitoring is the feasibility of its application to a broad spectrum of cells, including both proliferating and non-proliferating cells, as well as cells in tissues that are the first sites in which the genotoxic insult occurs. With the application of the comet assay to these various cell types, a better estimation of risk exposure can be made.

As previously mentioned, the most important details to consider with respect to a single cell suspension that is adequate for analysis using the comet assay include: The sampling protocol, sample storage, sample preparation, and adaptations of the comet assay. These aspects will be discussed in the present review.

## The comet assay in lens epithelial cells

The majority of studies using the comet assay in lens epithelial cells have been conducted in animals (Mitchell et al., 1998; Singh et al., 2002; Bannik et al., 2013; Liu et al., 2013; Aly and Ali, 2014), lymphocytes (Wolf et al., 2008; Liu et al., 2013) or human lens epithelial cell cultures (Lixia et al., 2006; Yao et al., 2008; Pierscionek et al., 2010, 2012; Gao et al., 2011; Liu et al., 2013) (Table 1).

**Table 1:** **Variations in comet assay protocols employing human lens epithelial cells**.

**Biomatrix**	**Lysis solution/Duration**	**Enzyme digestion**	**Electrophoresis buffer**	**Unwinding/Electrophoresis duration and V/cm**	**Neutralization buffer**	**Staining**	**Visualization and analysis of images**	**Number of comets**	**Measurement**	**References**
Lens	2.5 M NaCl, 100 mM Na_2_EDTA, 10 mM Tris HCL, 1% Na sarcosinate pH 10, 1% triton X-100, 10% DMSO/8 h		1 mM Na_2_EDTA, 300 mM NaOH, pH 13	20 min /20 min, 25 V	0.4 M Tris, pH 7.5	Ethidium bromide	Fluorescence microscope and CometScoreTM software	20 comets	% of DNA in the head and in the tail	Sorte et al., 2011
Lens	2.5 M NaCl, 100 mM Na_2_EDTA, 10 mM Tris HCL, 1% Na sarcosinate pH 10, 1% triton X-100, 10% DMSO/Overnight	30 min	1 mM Na_2_EDTA, 300 mM NaOH, pH 13	20 min/30 min, 1.4 V/cm, 300 mA	PBS	SYBR Gold	Comet Assay IV; Perceptive Instruments	50 comets	% of DNA in the tail	Øsnes-Ringen et al., 2013
Lens	2.5 M NaCl, 100 mM Na_2_EDTA, 10 mM Tris HCL, 1% Na sarcosinate pH 10, 1% triton X-100, 10% DMSO/Overnight		1 mM Na_2_EDTA, 300 mM NaOH, pH 13	20 min/20 min, 20 V/200 mA	0.4 M Tris, pH 7.5	Ethidium bromide	Fluorescence microscopy and CAPS software	50 comets	% Tail DNA and Olive tail moment	Zhang et al., 2014

When the lens epithelial cells of cataract patients are used directly (Sorte et al., 2011; Øsnes-Ringen et al., 2013; Zhang et al., 2014), the cells must be obtained and dissociated prior to their use in the comet assay.

### Sampling protocol and sample storage

In the study conducted by Sorte et al. (2011), lens epithelial cells from healthy controls were used after removal of the cornea and anterior capsule using a forceps. Continuous curvilinear capsulorhexis was performed through a clear corneal incision under local anesthesia in senile cataract patients. The anterior capsule was removed via viscoexpression through a clear corneal incision, and the anterior capsule was collected using forceps to avoid direct damage. After removal of the anterior capsule, the samples were maintained in minimum essential media. A single rhexis was placed in Eagle's Minimal Essential Medium containing 10% fetal bovine serum. Zhang et al. (2014) performed the same procedure as Sorte et al. (2011), with some modifications. A continuous curvilinear capsulorhexis was performed through a clear corneal incision under anesthesia. The anterior capsules were immediately removed and placed in Dulbecco's Modified Eagle's Medium containing 15% fetal bovine serum. The maximum amount of time that elapsed between sample collection and the initiation of processing was 30 min in both studies. Øsnes-Ringen et al. (2013) analyzed consecutive capsulotomy specimens obtained from age-related cataract patients. A clear corneal incision was made, viscoelastic material was introduced and the anterior capsule was extracted. The tissue samples were immediately placed in Dulbecco's Modified Eagle's Medium/F12 (DMEM/F12) containing 15% fetal bovine serum. The samples were analyzed either immediately or after 1 week incubation in the same medium at 37°C in the presence of 5% CO_2_.

### Comet assay sample preparation

Sorte et al. (2011) prepared a cell suspension using mechanical shaking of the capsule (in 50 μl of PBS) by hand for 10–15 min at 4°C to shed lens epithelial cells from the lens capsule, after which point the capsule was discarded. Øsnes-Ringen et al. (2013) and Zhang et al. (2014) prepared suspensions of single cells after pipetting the lens epithelium several times. After the capsule was discarded, the cell suspensions were centrifuged at 200 × g for 5 min at 4°C, the supernatants were discarded and the cells were resuspended in PBS.

### Comet assay

In the study conducted by Sorte et al. (2011), the comet assay was conducted according to the procedure developed by Singh et al. (1988), with a few modifications. Cells in the second agarose layer were embedded by mixing equal volumes of the cell suspension (50 μl) with 2% Low Melting Point Agarose (LMPA) instead of 80 μl of 1% LMPA and 20 μl of cell suspension. Zhang et al. (2014) embedded 50 μl of the cell suspension mixed with 100 μl of 0.75% LMPA onto slides that had been pre-coated with 0.75% Normal Melting Point Agarose (NMPA). Øsnes-Ringen et al. (2013) used 30 μl of the cell suspension mixed with 140 μl of 1% LMPA and 10 5 μl drops were placed onto a glass slide (that had been pre-coated with agarose and dried) as two rows of five (in the absence of coverslips) (Table 1).

#### Enzyme treatment

Of the three studies, only Øsnes-Ringen et al. (2013) used lesion-specific enzymes to detect specific types of DNA damage. After lysis, the slides were rinsed three times for 5 min each in enzyme buffer at 4°C. Using a silicone gasket and a plastic chamber (Shaposhnikov et al., 2010), each gel on the slide was isolated and incubated with 30 μl of buffer or enzyme (formamidopyrimidine DNA glycosylase, endonuclease III and T4 endonuclease V). Two gels were incubated with each of the solutions for 30 min at 37°C in a moist chamber (Table 1).

### Results

Sorte et al. (2011) detected prominent DNA migration in the majority of the cataractous lens epithelial cells, but not in the majority of the control subjects. DNA fragments in the tail of the comets displayed smearing, indicating that chemical damage had occurred.

Øsnes-Ringen et al. (2013) detected low levels of strand breaks, with mean values of DNA in the tails of 0.2 and 0.6% before and after cultivation, respectively.

Zhang et al. (2014) detected comets in the majority of the lens epithelial cells and lymphocytes of age-related cataract patients, as well as in some of the lymphocytes from the control patients, but comets were not detected in the majority of the lens epithelial cells that were derived from control patients. The researchers observed that DNA damage in lymphocytes was more severe than that in the corresponding lens epithelial cells from the same individuals, speculating that systemic, and local oxidative damage might affect each other.

### Discussion

DNA damage that was assessed using the comet assay in lens epithelial cells was mainly studied in the context of cataracts. This multifactorial pathogenesis is the major cause of blindness worldwide. Epidemiological, clinical and experimental studies indicate that UV radiation and oxidative stress are significant contributors to the development of lens opacities. In particular, DNA damage and cell death has been demonstrated in lens epithelial cells obtained from patients with cataracts. The low levels of strand breaks that were detected by Øsnes-Ringen et al. (2013) in the age related cataractous lens epithelium may be explained by patient selection and/or by the protocol that was used to obtain and process the samples. Similar considerations at respect to patient selection to those that apply to the variation in results reported from other groups may also apply to this study. The previously discussed investigations call for further studies of DNA damage in human lens epithelial cells from lenses with and without cataracts using the comet assay. In particular, such investigations may provide novel information regarding the mode of DNA damage progression *in vivo*, allow for informed *ex vivo* interventions to reduce damage and/or stimulate damage repair, and ultimately lead to clinical studies of prophylactic approaches.

## The comet assay in corneal cells

In corneal cells, studies using the comet assay have been conducted in animals (Rogers et al., 2004; Choy et al., 2005; Roh et al., 2008; Morkunas et al., 2011; Jester et al., 2012), lymphocytes of patients (Czarny et al., 2013) or human lens epithelial cell cultures (Wu et al., 2011; Ye et al., 2011, 2012).

When using the cornea directly (Haug et al., 2013; Lorenzo et al., 2013), the cells must be obtained and dissociated prior to use in the comet assay.

### Sampling protocol and sample storage

In the study conducted by Haug et al. (2013), the corneas were stored in Optisol GS at 4°C prior to transplantation and the remaining corneo scleral rims were acquired for the study. For the comet assay, 10 rims were used. Half of each rim was immediately processed for analysis, while the other half was transferred to Eye Bank Organ Culture (OC) for 1 week prior to analysis. This experimental design was selected to examine the effects of OC on tissue that had been previously stored in Optisol GS. Lorenzo et al. (2013) used human corneo-scleral tissue that was obtained from rings after penetrating keratoplasty and preserved in OC prior to use. The corneo-limbal rings were transferred to dishes containing DMEM/F12, in which the peripheral sclera and cornea were trimmed off. The rings were divided into 12 samples measuring approximately 2 × 2 mm. The samples were washed in Hanks Balanced Salt Solution in the absence of Ca^2+^ and Mg^2+^ at room temperature.

### Comet assay sample preparation

To obtain a single-cell suspension, Haug et al. (2013) removed the epithelium by scraping on ice before gentle pipetting and centrifuging at 200 × g for 5 min at 4°C. The cells were resuspended in PBS. Lorenzo et al. (2013) generated duplicate samples from each ring that were incubated at 37°C in a humid atmosphere containing 5% CO_2_ in pre-equilibrated 0.05% trypsin in HBSS containing 0.02% EDTA-_4_Na (in the absence of Ca^2+^ and Mg^2+^) for 1 or 3 h in either 250 μl or 3 ml of the solution using 96- or 6-well plates, respectively. At the end of the incubation period, enzyme activity was terminated by adding an equal amount of serum-containing growth medium (DMEM/F12). The cells were dispersed by gentle pipetting. The dissociated cells from each well in media/enzyme solution were transferred to tubes on ice.

### Comet assay/enzyme treatment

Haug et al. (2013) and Lorenzo et al. (2013) performed the comet assay according to the procedure developed by Azqueta et al. (2009), with some modifications. Haug et al. (2013) used lesion-specific enzymes to detect specific types of DNA damage. After lysis, the slides were rinsed in enzyme buffer at 4°C. Using a silicone gasket and a plastic chamber (Shaposhnikov et al., 2010), each gel in the slide was isolated and incubated with 30 μl of buffer or enzyme (formamidopyrimidine DNA glycosylase, endonuclease III, and T4 endonuclease V). The gels were incubated with each of the solutions for 30 min at 37°C in a moist chamber. Untreated lymphocytes were used as a negative control, and lymphocytes from healthy volunteers that had been treated on ice with 2 μM photosensitizer Ro 19-8022 plus visible light (a 500 W tungsten-halogen source at 33 cm) to induce 8-oxoGua were used as a positive control. The control cells were treated in the same manner as corneal epithelial cells, but were incubated with only enzyme buffer or FPG (Table 2).

**Table 2:** **Variations in comet assay protocols employing human corneal cells**.

**Biomatrix**	**Lysis solution/Duration**	**Enzyme digestion**	**Electrophoresis buffer**	**Unwinding/Electrophoresis duration and V/cm**	**Neutralization buffer**	**Staining**	**Visualization and analysis of images**	**Number of comets**	**Measurement**	**References**
Corneal	–/Overnight		–	20 min/20 min, 20 V aprox. 300 mA	PBS 10 min	SYBR Gold	Comet Assay IV; Perceptive Instruments	50 comets	% of DNA in the tail	Haug et al., 2013
					H_2_O 10 min					
Corneal	2.5 M NaCl, 100 mM Na_2_EDTA, 10 mM Tris HCL, 1% Na sarcosinate pH 10, 1% triton X-100, 10% DMSO/1 h		1 mM Na_2_EDTA, 300 mM NaOH, pH 13	20 min/20 min, 1.3 V/cm	PBS 10 min	SYBR Gold	Visually classified using fluorescence microscopy	100 comets	Overall score ranging from 0 to 400 units	Lorenzo et al., 2013
					H_2_O 10 min					

### Results

Haug et al. (2013) found that the levels of strand breaks were low in cold-stored tissues. Enzyme-sensitive sites were generally not increased by much in OC, with the exception of certain samples that displayed substantial increases in Endo III-sensitive sites (oxidized pyrimidines); marked increased were observed in 3 of the 10 samples, while the levels of FPG-sensitive sites were similar in the two groups. The levels of T4 endo V sites increased.

In the study conducted by Lorenzo et al. (2013) using trypsin-EDTA, DNA damage was observed in the form of strand breaks, regardless of the volume of enzyme solution and the duration of incubation. A trend toward increased damage was observed when using 3 ml compared to 250 μl. Increasing the incubation time from 1 to 3 h did not consistently increase the levels of strand breaks.

### Discussion

Previously, studies of human cells using the comet assay have generally focused on blood cells or cultivated cells.

Little information is available regarding the amounts of molecular damage inflicted upon essential molecular constituents, including DNA, by the different protocols. We report that the comet assay may yield valuable information regarding the amounts and types of DNA damage in such tissues. However, in contrast to blood cells, the cells in these tissues must be dissociated prior to analysis using the comet assay, and such methods may induce various types and amounts of DNA damage.

For the *ex-vivo* storage, culture and engineering of tissues for transplant purposes, one main challenge is to provide tissues in which the individual cells harbor minimal amounts of molecular damage, in particular DNA damage. The study conducted by Haug et al. (2013) using the corneal epithelium demonstrated that cells may be dissociated from the cornea using mechanical procedures. Subsequent investigations using the comet assay provided information regarding the levels and types of DNA damage under different storage and incubation conditions. Lorenzo et al. (2013) demonstrated that dissociation of corneo-limbal epithelial cells using trypsin-EDTA, a procedure that is commonly used to initiate cultures for the *ex-vivo* engineering of transplantable tissues, is associated with DNA damage.

The protocols used for tissue storage and culture and the *ex-vivo* engineering of tissues for transplant purposes differ between clinics, and various types of nutrient solutions and incubation conditions are commonly used. In addition, cells may be dissociated from the original tissue using enzyme solutions prior to tissue engineering and seeded on a substrate for propagation. Certain protocols call for the positioning of tissue samples directly on the substrate. On such substrates, novel tissue is generated by cells that migrate from the tissue of origin out onto the substrate.

The studies outlined above demonstrate that the comet assay may provide crucial information regarding the integrity of the DNA in such tissues. Such information is of significant value in research aimed at improving *ex-vivo* conditions and the quality of tissues destined for transplantation.

## The comet assay in exfoliated tear duct cells

The search for relevant target cells for human monitoring has revealed the potential use of exfoliated tear duct epithelial cells in the comet assay. To date, only one study has applied these cells during comet assay monitoring (Rojas et al., 2000). The main lachrymal gland serves to keep the eye surface clean, using tears to clear the eye of desquamated cells, particles, and diluting gasses or liquids. The gland is located beneath the conjunctiva on the upper lateral margin of the orbit and drains into the upper fornix of the conjunctiva via a series of approximately 10 small ducts. The cells that desquamate into the tear film are those from the cornea, which is covered by stratified, squamous, non-keratinized epithelium with a basal cell layer that gives rise to five to six superficial layers.

### Sampling protocol and sample storage

A total of 20 μl of tears were obtained using a 20 μl capillary tube from the inner nasal angle of the right eye while nasal brushing was performed, stimulating the olfactory bulb. The samples were maintained in the capillary tubes at room temperature prior to performing the comet procedure.

### Comet assay sample preparation

Epithelial cells contained in a tear film, which served as a physiological solution, did not require special preparation. The samples contained in the capillary tubes were pushed using a gum bulb into a microtube to be mixed with LMPA (0.5%) (Table 3).

**Table 3:** **Overview of the comet assay protocol employing tear duct epithelial cells**.

**Biomatrix**	**Lysis solution/duration**	**Enzyme digestion**	**Electrophoresis buffer**	**Unwinding/Electrophoresis duration and V/cm**	**Neutralization buffer**	**Staining**	**Visualization and analysis of images**	**Number of comets**	**Measurement**	**Reference**
Tear duct	2.5 M NaCl, 100 mM Na_2_EDTA, 10 mM Tris Base, pH 10, 1% Triton X-100, 10% DMSO/1 h		1 mM Na_2_EDTA, 300 mM NaOH, pH 13	20 min/20 min, 0.8 V/cm	0.4 M Tris pH 7.5	Ethidium bromide	Scaled ocular	50 comets	Tail length Microns	Rojas et al., 2000

### Comet assay

The alkaline procedure was conducted by pipetting 75 μl of the cell mixtures (tears and LMPA) onto a slide that had been pre-coated with 180 μl of normal agarose and immediately covered with a coverglass to form a microgel, allowing the agarose to jellify. A third LMP (0.5%) agarose layer was added. The slides were immersed in lysis solution (pH 10) for 1 h. The DNA was allowed to unwind for 20 min in electrophoresis buffer, and electrophoresis was then conducted at 0.8 V/cm for 20 min (Table 3).

### Results

The authors of the study only analyzed 25 nucleoids per slide in duplicate, suggesting a low quantity of cells in 20 μl of tear film. DNA damage increased in the tear duct epithelial cells of individuals exposed to urban atmospheres with high ozone concentrations. Because this is the only study that was conducted using this type of cell, comparison of the procedures is not possible.

### Discussion

The study presented data regarding the use of exfoliated tear duct epithelial cells for monitoring. This cell type presents various advantages for monitoring, as follows: The cells can be acquired using minimally invasive procedures; sufficient cells are present in only one tear drop; and these cells are relevant to genotoxicity studies involving cosmetic products, airborne carcinogens, and all agents that may come in contact with the eyes (Rojas et al., 2000).

## The comet assay in buccal cells

Evaluation of DNA damage in buccal epithelial cells may provide a biomarker of early damage in target tissues. This type of cells has been employed principally in human studies through non-invasive methods and is easily applied as a biomarker in biomonitoring studies in a similar manner to micronuclei.

These cells must be directly obtained from the oral cavity and dissociated prior to use in the comet assay.

### Sampling protocol and sample storage

In the studies conducted by Rojas et al. (1996), Valverde et al. (1997), Eren et al. (2002), and Beričević et al. (2012), buccal cells were obtained after the use of mouth wash by scraping the internal part of the cheek with a wood or plastic stick and were added to RPMI-1640 medium during transportation prior to being rapidly processed. Faccioni et al. (2003) collected buccal mucosal cells by gently brushing the internal portion of the cheeks using an interdental brush after washing out the mouth many times with tepid water. The brushes were stirred in 5 ml of PBS (pH 7.4). Similarly Szeto et al. (2005), Jayakumar et al. (Jayakumar and Sasikala, 2008), and Mondal et al. (2011) used soft bristle toothbrushes to collect buccal cells by scraping the inside of the cheeks after rinsing the mouth with distilled water. The toothbrushes were then agitated in 30 ml of cold PBS. Ursini et al. (2006) and Cavallo et al. (2006, 2009) also collected exfoliated buccal cells after the subjects had rinsed their mouths with water by scraping the interior of the cheeks with a toothbrush. They suspended the cells in 25 ml of Titenko-Holland buffer containing 0.01 M Tris-HCl, 0.1 M EDTA and 0.02 M NaCl (pH 7), and immediately sent the cells to the laboratory to perform the assay. Westphalen et al. (2008) collected the cells by gentle brushing of the inside portion of the lower lip with a cytobrush after washing the mouth out several times with tepid distilled water. The brushes were stirred in 20 ml of PBS. Sudha et al. (2011) and Eshkoor et al. (2011, 2013) obtained the cells by gently rubbing the inside of both cheeks with an extra soft toothbrush for 1 min. The brushes were then rinsed in a tube containing 30 ml of saline before finally being washed with PBS (pH 7.4). Pal et al. (2012) obtained the cells using oral brushing after the subjects had washed their mouths with normal saline (a 0.9% NaCl solution). The collected samples were maintained in PBS. Visalli et al. (2013) obtained the oral mucosal cells by scraping the cheeks with a moist wooden spatula. Prior to scraping, the subjects rinsed their mouths with saline.

### Comet assay sample preparation

Various studies generated suspensions of cells that were immersed in RPMI-1640 via centrifugation over a range of 1–10 min at 800–6000 rpm (Rojas et al., 1996; Jayakumar and Sasikala, 2008; Westphalen et al., 2008; Mondal et al., 2011; Sudha et al., 2011). Similarly, Szeto et al. obtained pellets and resuspended them in 100 μl of PBS. Additional procedures were also reported (Faccioni et al., 2003), in which the cell suspensions were centrifuged, suspended in PBS, and filtered through polyamide gauze (with a 100 μm mesh opening). The filtrates were pelleted using centrifugation and resuspended in RPMI-1640. Beričević et al. (2012) centrifuged the cell suspension for 3 min at 3200 rpm and resuspend it in PBS (pH 7.4), after which point cell viability was determined and one aliquot was immediately resuspended in a chilled buffer at pH 7.5 (containing 0.075 M NaCl and 0.024 M Na_2_ EDTA). The cells were macerated on ice for 2 min. Visalli et al. (2013) reported that after 1 h, the exfoliated cells were processed at 800 × g for 3 min and the pellets were suspended in 40 μl of PBS. The number of epithelial cells, on average, ranged from 1 to 2 × 10^6^/ml, which equated to 40,000–80,000 cells per subject. However, reports in which exfoliated buccal cells were washed twice in PBS and then suspended in approximately 100 μl of the same buffer in the absence of centrifugation have also been made (Cavallo et al., 2006, 2009; Ursini et al., 2006). In addition, it is important to note that certain groups did not provide data regarding sample preparation because the use of specific kits or details of this manner were not included in the publications(Valverde et al., 1997; Eren et al., 2002; Eshkoor et al., 2011, 2013; Pal et al., 2012).

### Comet assay/enzyme treatment

Only four reports used the original three agarose layers containing the same percentages that were reported by Singh et al. (1988) (i.e., 0.5%) (Rojas et al., 1996; Valverde et al., 1997; Ursini et al., 2006). The majority of reports employed only two agarose layers containing volumes ranging from 70 to 100 μl of LMPA and NMPA, and the percentage of agarose used ranged from 0.7 to 1%.

The majority of the studies that were conducted to determine DNA damage in buccal epithelial cells used the alkaline comet assay according to the procedure developed by Singh et al. (1988), with various modifications. Only the studies that employed the modifications outlined by Szeto et al. (Szeto et al., 2005) performed neutral comet assays (pH 9.1) (Jayakumar and Sasikala, 2008; Mondal et al., 2011; Pal et al., 2012) (Table 4).

**Table 4:** **Variations in comet assay protocols employing human buccal cells**.

**Biomatrix**	**Lysis solution/Duration**	**Enzyme digestion**	**Electrophoresis buffer**	**Unwinding/Electrophoresis duration and V/cm**	**Neutralization buffer**	**Staining**	**Visualization and analysis of images**	**Number of comets**	**Measurement**	**References**
Buccal	2.5 M NaCl, 100 mM Na_4_EDTA, 10 mM Tris HCL, 1% Na sarcosinate pH 10, 1% triton X-100, 10% DMSO/48 h	2 h Proteinase K 140 μl (10 mg/ml) 37°C 2.5 M NaCl, 100 mM Na_4_EDTA, 10 mM Tris HCL, 1% Na sarcosinate pH 10, 1% triton X-100, 10% DMSO	1 mM Na_2_EDTA, 300 mM NaOH, pH 13	20 min/20 min, 25 V/300 mA	0.4 M Tris, pH 7.5	Ethidium bromide	Scaled ocular	100 comets	Tail length Microns	Rojas et al., 1996
Buccal	2.5 M NaCl, 100 mM Na_4_EDTA, 10 mM Tris HCL, 1% Na sarcosinate pH 10, 1% triton X-100, 10% DMSO/24 h	1 h Proteinase K 100 μl (10 mg/ml) 37°C 2.5 M NaCl, 100 mM Na_4_EDTA, 10 mM Tris HCL, 1% Na sarcosinate pH 10, 1% triton X-100, 10% DMSO	1 mM Na_2_EDTA, 300 mM NaOH, pH 13	20 min/20 min, 25 V/300 mA	0.4 M Tris, pH 7.5	Ethidium bromide	Scaled ocular	100 comets	Tail length Microns	Valverde et al., 1997
Buccal	2.5 M NaCl, 100 mM EDTA, 10 mM Tris HCL, 1% Na sarcosinate pH 10, 1% triton X-100, 10% DMSO 1 h	2 h Proteinase K 140 mg/ml 37°C	1 mM Na_2_EDTA, 300 mM NaOH, pH 13	40 min/40 min	0.4 M Tris, pH 7.5	Ethidium bromide	Visually classified using fluorescence microscopy	100 comets	Cell percentage in three categories	Eren et al., 2002
Buccal	-/Overnight		-	25 min/20 min, 25 V/300 mA (0.86 V/cm)		Ethidium bromide	Comet Assay II; Perceptive instruments	100 comets	Tail length, % Tail DNA, and Olive tail moment	Faccioni et al., 2003
Buccal	30 min 50 μl (0.25%Trypsin, 1 mM EDTA in Hanks balanced salt solution)	1 h Proteinase K (1 mg/ml).	1 mM NaOH, 1 mM EDTA, pH 9.1	20 min/18 min, 12 V constant	0.4 M Tris, pH 7.5	Ethidium bromide	Komet 3.0 software	Two gels per donor	Tail DNA %	Szeto et al., 2005
		1 h conventional lysis (2.5 M NaCl, 100 mM Na_2_EDTA, 10 mM Tris HCL, 1% Na sarcosinate pH 10, 1% triton X-100, 10% DMSO)								
Buccal	2.5 M NaCl, 100 mM Na_2_EDTA, 10 mM Tris HCL, 1% Na sarcosinate pH 10, 1% triton X-100, 10% DMSO/1 h		1 mM Na_2_EDTA, 300 mM NaOH, pH 13	20 V/300 mA, 20 min	0.4 M Tris, pH 7.5	Ethidium bromide	Image analyzer Delta Sistemi	50 comets	Tail moment	Ursini et al., 2006
Buccal	2.5 M NaCl, 100 mM Na_2_EDTA, 10 mM Tris HCL, 1% Na sarcosinate pH 10, 1% triton X-100, 10% DMSO/1 h	30 min FPG (1 μg/ml)	1 mM Na_2_EDTA, 300 mM NaOH, pH 13	40 min/30 min, 25 V/300 mA	0.4 M Tris, pH 7.5	Ethidium bromide	Image analyzer Delta Sistemi	50 comets	Tail moment	Cavallo et al., 2006
Buccal	-/Overnight	-	-	25 min/20 min, 25 V/300 mA (0.86 V/cm)	-	Silver nitrate	Light microscope	50 comets	Overall score of between 0 and 400 arbitrary units	Westphalen et al., 2008
Buccal	30 min 50 μl (0.25%Trypsin, 1 mM EDTA in Hanks balanced salt solution)	1 h Proteinase K (1 mg/ml).	0.01 M NaOH 1 mM EDTA, pH 9.1	20 min/18 min, 12 V(constant)	0.4 M Tris, pH 7.5	Ethidium bromide	Komet 5.5 software		Tail DNA % Tail length Olive tail moments	Jayakumar and Sasikala, 2008
		1 h conventional lysis (2.5 M NaCl, 100 mM Na_2_EDTA, 10 mM Tris HCL, 1% Na sarcosinate pH 10, 1% triton X-100, 10% DMSO)								
Buccal	2.5 M NaCl, 100 mM Na_2_EDTA, 10 mM Tris HCL, 1% Na sarcosinate pH 10, 1% triton X-100, 10% DMSO/1 h		1 mM Na_2_EDTA, 300 mM NaOH, pH 13	20 V/300 mA, 20 min/20 min	0.4 M Tris, pH 7.5	Ethidium bromide	Image analyzer Delta Sistemi	50 comets	Tail length Tail DNA % Tail moment	Cavallo et al., 2009
Buccal	-/1 h	-	-	-	-	Ethidium bromide	Image analysis s system	100 comets	Tail length microns	Sudha et al., 2011
Buccal	30 min 50 μl (0.25%Trypsin, 1 mM EDTA in Hanks balanced salt solution)	1 h Proteinase K (1 mg/ml).	0.01 M NaOH, 1 mM EDTA, pH 9.1	20 min/18 min, 12 V constant	0.4 M Tris, pH 7.5	Ethidium bromide	Komet 3.0 software	Two gels per donor	Tail DNA %	Mondal et al., 2011
		1 h conventional lysis (2.5 M NaCl, 100 mM Na_2_EDTA, 10 mM Tris HCL, 1% Na sarcosinate pH 10, 1% triton X-100, 10% DMSO)								
Buccal	Trevigen lysis/1 h	-	-	45 min/10 min, 1 V/cm^−1^	-	SYBR Green	Tri Tek comet score 1.5	-	Tail length	Eshkoor et al., 2011
Buccal	2.5 M NaCl, 100 mM Na_2_EDTA, 10 mM Tris HCL, 1% Na sarcosinate pH 10, 1% triton X-100, 10% DMSO/72 h		1 mM Na_2_EDTA, 300 mM NaOH, pH 13	10 min/16 min, 300 mA (0.66 V/cm)	0.4 M Tris, pH 7.5	Ethidium bromide	Comet assay IV software	100 comets	Tail DNA % Tail length	Beričević et al., 2012
Buccal	30 min 50 μl (0.25%Trypsin, 1 mM EDTA inn Hanks balanced salt solution)	1 h Proteinase K (1 mg/ml).	10 mM NaOH, 1 mM EDTA, pH 9.1	10 min/20 min, 30 V constant	0.4 M Tris, pH 7.4	Ethidium bromide	Komet 5.5 software	100 comets	Tail DNA % Olive tail moment	Pal et al., 2012
Buccal	Trevigen lysis/1 h	-	-	45 min/10 min, 1 V/cm^−1^	-	SYBR Green	Tri Tek comet score 1.5	-	Tail length	Eshkoor et al., 2013
Buccal	2.5 M NaCl, 100 mM Na_2_EDTA, 10 mM Tris HCL, 1% Na sarcosinate pH 10, 1% triton X-100, 10% DMSO/ Overnight	Proteinase K 8 μl (10 mg/ml) 15 min, 40 °C	1 mM Na_2_EDTA 300 mM NaOH pH 13	20 min/40 min, 300 mA 25 V (0.86 V/cm)		Ethidium bromide	Image analysis system CASP	50 comets	Tail DNA %	Visalli et al., 2013

However, the principal challenge in using this cell type is the cellular modifications that occur in the epithelium; it is thus important to take the enzymatic procedure that is employed during the lysis process to obtain free DNA that can respond to the electrophoretic field in to account. Various studies only used the lysis protocol that was originally proposed by Singh et al. (1988) (0.1 M EDTA, 2.5 M NaCl, 0.01 M Tris, and 1% N-laurylsarcosine, pH 10, with the fresh addition of 1% Triton X-100 and 10% DMSO) (Faccioni et al., 2003; Cavallo et al., 2006, 2009; Ursini et al., 2006; Westphalen et al., 2008; Sudha et al., 2011; Beričević et al., 2012). Certain studies reported the use of lysis conditions that corresponded to those of a specific kit (Eshkoor et al., 2011; Sudha et al., 2011), while other studies utilized a combination of different lysis conditions. However, all of the studies employed proteinase K (broad-spectrum serine protease) digestion under optimal conditions during lysis (Rojas et al., 1996; Valverde et al., 1997; Eren et al., 2002; Szeto et al., 2005; Jayakumar and Sasikala, 2008; Mondal et al., 2011; Pal et al., 2012; Visalli et al., 2013) using the procedure that was first outlined by Szeto et al. (Faccioni et al., 2003), in which proteinase K lysis is achieved using trypsin/EDTA digestion (Jayakumar and Sasikala, 2008; Mondal et al., 2011; Pal et al., 2012) (Table 4).

In the reviewed studies using buccal epithelial cells, only the Cavallo et al. (2006) study used FPG to detect oxidative DNA lesions (Table 4).

### Results

The study conducted by Rojas et al. (1996) employed enzymatic lysis enrichment to compare DNA damage between buccal epithelial cells that were derived from smokers and non-smokers; in spite of the fact that the study employed a small number of subjects; the comet assay was found to be suitable for use in this cell type. In the study conducted by Valverde et al. (1997), DNA damage induced by air pollution in Mexico City was compared in three different cell types, demonstrating that lysed buccal epithelial cells with proteinase K enrichment are suitable for use in comet analyses; however, differences between the exposure groups were not detected. The same protocol was recently employed by Visalli et al. (2013) to determine that subjects with restorative dental fillings (both amalgams and resin-based fillings) displayed genotoxic damage in the oral mucosa. The study conducted by Eren et al. (2002) examined the effects of chlorhexidine in blood and buccal epithelial cells and found that the comet assay in combination with lysis enrichment was able to identify damaged cells with greater sensitivity than the determinations of damage that were conducted in blood cells that had been obtained from the same subjects. In contrast, the study conducted by Westphalen et al. (2008), in which the comet assay was performed in buccal cells in the absence of modifications, did not detect orthodontic appliance-induced DNA damage after 10 days. However, Faccioni et al. (2003) and Beričević et al. (Eren et al., 2002) determined that nickel and cobalt released from fixed orthodontic appliances can induce DNA damage in oral mucosal cells in the absence of modifications to the Singh et al. procedure (Singh et al., 1988). In a similar manner, the studies conducted by Ursine et al. (Mondal et al., 2011) and Cavallo et al. (2009), in which changes to the protocol were not made, obtained negative comet assay results in buccal epithelial cells in healthcare workers handling antineoplastic drugs. In contrast, other studies performed in the absence of lysis modification obtained increased DNA migration using the comet assay in buccal epithelial cells to determine the effects of exposure to polycyclic aromatic hydrocarbons (PAHs) (Cavallo et al., 2006, 2009) among metal welders (Sudha et al., 2011) and mechanical workshop employees (Eshkoor et al., 2011, 2013). The study was performed according to the major procedural changes that were outlined by Szeto et al. (2005), and the modifications enabled the application of the comet assay in human biomonitoring and nutritional studies. This group proposed that successful lysis can be achieved using 0.25% trypsin for 30 min followed by proteinase K (1 mg/ml) treatment for 1 h and electrophoresis at a neutral pH (0.01 M NaOH and 0.001 M EDTA, pH 9.1). They induced H_2_O_2_-mediated DNA damage in a dose-dependent manner and observed Trolox (a water–soluble analog of vitamin E) protection. They also demonstrated that *in situ* exposure to antioxidant-rich green tea diminished DNA strand breaks. The same procedure was applied in buccal epithelial cells by Jayakumar and Sasikala (2008), who observed increased levels of DNA damage among jewelry workers and demonstrated the synergistic ability of cigarette smoking to induce DNA damage. Mondal et al. (2011) also employed the modifications outlined by Szeto et al. (2005) to demonstrate the induction of buccal epithelial DNA damage in women chronically exposed to biomass smoke. The report by Pal et al. (2012), which employed the same procedure, demonstrated that tobacco-associated DNA damage in the oral mucosa was decreased following the regular consumption of black tea.

### Discussion

Buccal epithelial cells may be considered to be short-lived cells (with renewal of 10–14 days) due to their continued renewal, while in comparison, peripheral blood lymphocytes may be considered to be longer-lived cells. Therefore, the presence of buccal cells with comet-like appearances is indicative of recent exposure to various substances. This consideration may explain the higher levels of DNA damage that were observed in buccal epithelial cells after exposure to agents that came into direct contact with the oral mucosa, the molecular mechanisms underlying which are closely related to the oxidative DNA damage that is induced by air pollutants and the inflammation that is triggered by the use of orthodontic apparatus.

The use of buccal epithelial cells to determine genotoxicity using the comet assay according to the procedure outlined by Singh et al. (1988) was limited by the inability to obtain free nucleoids. Originally, the enrichment of lysis solution with proteinase K was proposed to eliminate cellular- and nuclear-associated proteins to obtain nucleoids that would migrate in the electric field during alkaline electrophoresis (Rojas et al., 1996; Valverde et al., 1997; Eren et al., 2002). The previously mentioned studies employed RMPI-1640 as a vehicle to maintain buccal epithelial cells, and used similar concentrations of proteinase K during lysis. However, after these studies, confidence in the procedure that was used to conduct the comet assay with buccal epithelial cells began to decrease due to inconsistencies in the sampling procedure, which justified the use of protocols that did not employ lysis modifications in contrast to studies that incorporated many modifications to the procedure (Rojas et al., 1996; Valverde et al., 1997; Eren et al., 2002; Faccioni et al., 2003; Szeto et al., 2005; Cavallo et al., 2006; Ursini et al., 2006; Jayakumar and Sasikala, 2008; Westphalen et al., 2008; Cavallo et al., 2009; Eshkoor et al., 2011; Mondal et al., 2011; Sudha et al., 2011; Beričević et al., 2012; Pal et al., 2012; Eshkoor et al., 2013). However, it is important to note that the studies conducted by Szeto et al. (2005), Jayakumar and Sasikala (2008), Mondal et al. (2011), and Pal et al. (2012) revealed different types of DNA fragments due to the use of neutral conditions followed by the unwinding and electrophoresis procedures. Thus, these results are only comparable to those of studies that conducted the same procedure (Table 4).

Future reports should include images that support the use of the comet assay in buccal epithelial cells, as well as details regarding sampling and the manner in which the cell suspension was handled prior to lysis. The use of a non-invasive method to obtain buccal epithelial cells and the potential to determine genotoxicity in cells that come in to direct contact with the potential insult are important aspects that are required to validate the use of a procedure during the alkaline comet assay.

## The comet assay in nasal cells

The search for relevant target cells that can be used to study the genotoxic effects of xenobiotics has increased over the past few years. In this context, nasal tissue cells are the first to come in contact with environmental xenobiotics. Exfoliated mucosal cells have been postulated to have predictive value for the detection of carcinogens because 90% of human tumors are of epithelial origin (Cairns, 1975). The comet assay has been examined as a suitable and rapid screening method to determine chemical substance-induced DNA damage in human nasal mucosal cells (Pipkorn et al., 1988).

### Sampling protocol and sample storage

The studies performed using this cell type typically apply one of three different sampling protocols. The first sampling protocol is the classical method of obtaining cells from the lower edge of both lower nasal turbinates using a disposable nylon brush or cytobrush under direct visual inspection; this method is neither painful nor invasive (Pipkorn et al., 1988; Calderon-Garcidueñas et al., 1996). The nasal epithelium that was obtained using this procedure was immediately immersed in 1 ml of cold RPMI-1640 medium. All of the samples were collected at the same time and rapidly processed. This procedure was used by Calderon-Garcidueñas et al. (Valverde et al., 1997; Calderon-Garcidueñas et al., 1999; Glück and Gebbers, 2000; Kleinsasser et al., 2001; Tisch et al., 2002, 2005; Fortoul et al., 2003a,b, 2004, 2010; Gosepath et al., 2003; Pacini et al., 2003; Koreck et al., 2007; Hölzer et al., 2008; Ginzkey et al., 2012).

Another sampling protocol obtained the cells via nasal epithelial biopsies (Kleinsasser et al., 2001; Gosepath et al., 2003; Tisch et al., 2005; Hölzer et al., 2008; Ginzkey et al., 2012). Following blood clot removal and the proteolytic separation (50 mg of protease, 10 mg of hyaluronidase, and 10 mg of collagenase) of mucosal cells, Tisch et al. (Gosepath et al., 2003) adjusted the cell number to 1 × 10^6^ cells/ml in Joklik medium. In a study conducted by Gosepath et al. (Sassen et al., 2005), after harvesting and mincing the biopsy specimens, the specimens were trypsinated in pronase for 24 h at 4°C and digested for 15 min at 37°C. The cells were then washed in a phosphate buffer solution and centrifuged. In a study conducted by Tisch et al. (Hölzer et al., 2008), the tissue was incubated with a proteolytic enzyme solution in a shaking water bath at 37°C for 60 min. Fetal calf serum (FCS) was added to avoid uncontrolled enzyme activity. Meanwhile, Hölzer et al. (Reiter et al., 2009) performed mucosal cell disintegration via enzymatic digestion (50 mg of protease, 10 mg of hyaluronidase, and 10 mg of collagenase in 10 ml Ham's F12) for 30 min. Digestion was terminated by centrifugation (10 min at 276 × g), removal of the enzyme solution, and resuspension of the cells in culture medium.

Recently, Ginzkey et al. (Baumeister et al., 2009a) performed another protocol, in which nasal mucosal specimens were obtained during human nasal passage surgery. The nasal mucosa was separated from the bone and connective tissue via enzymatic digestion in a manner that differed from that used in other studies [100 μl of enzyme mix containing 0.1 g of protease and 1.0 mg of DNase dissolved in 10 ml of phosphate buffered saline were prepared using 9 ml of Airway Epithelial Growth Medium (AEGM)]. The specimens were incubated with enzymes for 24 h on a shaker at 4 °C. After terminating the enzymatic reaction with FCS, the cell suspension was filtered through sterile gauze, and washed twice with PBS. Cell number and viability were assessed using the trypan blue exclusion test.

A third method involved the sophisticated generation of 3D miniorgan cultures of human inferior turbinate epithelia (MOCs) from nasal biopsies (Baumeister et al., 2009b; Hackenberg et al., 2010, 2011; Koehler et al., 2010, 2013). Following immediate transport to the laboratory, the cells were minced in 25 × 5 mm pieces and washed three times in Bronchial Epithelial Growth Medium (BEGM) and placed in 24-well plates(one fragment per well). The wells were coated with 0.75% agar noble that had been dissolved in Dulbecco's Modified Eagle Medium containing 10% FCS and non-essential amino acids, streptomycin, and amphotericin B. MOCs floated in 250 μl of BEGM per dish at 37°C, 5% CO_2_, and 100% relative humidity. Adhesion to the dish surface was prevented using agarose. BEGM was renewed every other day, and the multiwell plates were replaced on days 7 and 9 to renew the agarose. After 5 days, the initial mucosal fragments were completely coated with partly ciliated epithelium (Baumeister et al., 2009a,b; Hackenberg et al., 2011; Koehler et al., 2010, 2013). In Sassen et al. (2005) and Hackenberg et al. (2010), a similar protocol was performed, with the difference being the use of Airway Epithelial Cell Growth Medium (AECGM) in place of BEGM. In addition, Buehrlen et al. (Hackenberg et al., 2011) used Bronchial Epithelial Basal Medium (BEBM) in place of BEGM. Both authors used penicillin in place of amphotericin B.

Recent studies conducted by Koehler et al. (2010, 2013) and Hackenberg et al. (2010, 2011) utilized a new biopsy handling protocol. Upon receipt, the specimens were cleaned of blood and cartilage by washing in Minimum Essential Medium to isolate the epithelial cells from the specimens, and the cells were then incubated for 24 h in a mixture of 10 ml MEM that had been supplemented with 0.1 mg/ml of protease XIV, 1 mg/ml of DNAse DN25 and antibiotics (0.05 mg/ml of gentamicin, 100 U/ml of penicillin containing 1 μg/ml of streptomycin, 0.250 U/ml of amphotericin B and 2 ml of glutamine). After 24 h, the enzyme activity was terminated by adding 5 ml of FCS. The cells were then scratched from the specimen with a scalpel and poured into a dish. This cell suspension was centrifuged at 500 × g for a duration of 5 min. The cell pellets were resuspended in 1 ml of AECGM that had been supplemented with antibiotics (100 U/ml of penicillin and 1 μg/ml of streptomycin). Cell viability was assessed by vital staining with 0.4% trypan blue, and the number of cells was determined using a light microscope. The human nasal cells were cultured on porous membrane inserts (0.4 μm Corning® Transwell polycarbonate membrane inserts; 12 mm diameter). The porous membrane inserts were covered with 150 μl of collagen I (66 ng/ml), incubated for 3 h at 37°C in a humidified incubator and then stored at 4°C until use. A total of 10^4^ epithelial cells were cultured in the BEGM suspension and pipetted onto single membrane inserts. Additional media was added until a minimum of 1.5 ml of BEGM was apical to the membrane and 2 ml was present beneath the membrane in the well. The plates containing the membranes were cultured at 37°C in a humidified incubator in the presence of 5% CO_2_. The cells attached to the membrane within 2–3 h. The media was changed every 48 h and the membranes were washed with 2 ml of PBS during the media exchange. After reaching 70–80% confluence on day 7, the media that was apical to the membrane was removed and nutrition was provided to the cells by adding 1.3 ml of BEGM per insert under the membrane. At this point, the cultures achieved air-liquid interface conditions, which were maintained from days 7 to 14 to stabilize the culture conditions. Media exchange beneath the membrane and apical rinsing of the membranes with 2 ml of PBS were carried out three times per week.

### Comet assay sample preparation

The nasal epithelium that was obtained using the cytobrush was immediately immersed in 1 ml of cold RPMI-1640 medium. The nasal samples were easily dispersed into single cells by gently shaking the glass tubes. The single nasal cell suspension volume was then adjusted to 50,000 cells/50 μl of medium (Calderon-Garcidueñas et al., 1996, 1999; Rojas et al., 1996; Fortoul et al., 2003a,b, 2004, 2010). Concurrently, Pacini et al. (Fortoul et al., 2003a) soaked and shook the nylon brush in 2 ml of ice-cold, oxygenated (5% CO_2_) minimum essential medium that had been supplemented with 10% FCS. The released cells were maintained on ice and in the dark for no longer than 2 h and were subsequently centrifuged at 250 × g at 4°C for 10 min. The resulting pellets were resuspended in 100 μl of ice-cold medium, and the nasal cell suspension volume was adjusted to prepare the comet slides.

In contrast, the nasal cells that were obtained from biopsies were treated after this exposure period; the viability of the cultures was examined using trypan blue and the cultures were then centrifuged for 10 min at 400 rpm. Once the obtained cell pellets had been resuspended in 1 ml of fresh medium, the final cell suspension was available (Kleinsasser et al., 2001; Tisch et al., 2005; Hölzer et al., 2008). It is also important to mention that some of the groups did not provide information regarding sample preparation (Gosepath et al., 2003; Ginzkey et al., 2012).

Following xenobiotic treatment, the MOCs were enzymatically digested by incubation for 45 min at 37°C with collagenase P (1 mg/ml), hyaluronidase that had been isolated from bovine testes (1 mg/ml) and pronase E (5 mg/ml) that had been dissolved in BEGM. The enzymes were neutralized using FCS, and the cells were washed twice in cold PBS.

### Comet assay/enzyme treatment

Comet slide preparation varied with respect to agarose layer number, agarose percentage, and volume. In spite of these differences, similarities in the nasal cell procedures were also identified; for instance, nearly all of the studies that obtained samples using a cytobrush applied three agarose layers (0.5% NMPA was used for the first layer, followed by 0.5% LMPA for the second and third layers) (Valverde et al., 1997; Fortoul et al., 2003b, 2004; Koreck et al., 2007). Pacini et al. (Fortoul et al., 2003a) employed three agarose layers, all of which consisted of LMPA; however, information regarding the concentrations used was not reported. Gluck and Gebbers (Pacini et al., 2003) also omitted these details. Calderon-Garcidueñas (Calderon-Garcidueñas et al., 1999; Glück and Gebbers, 2000) reported the use of two agarose layers (0.5% NMPA followed by LMPA), without specifying the concentrations used. Studies in which the nasal epithelial cells were obtained from biopsies typically utilized three layers, with the exception of Gosepath (Tisch et al., 2005), which only reported the use of one LMPA layer, and Ginzkey (Kleinsasser et al., 2001), which reported the use of two layers(1.5% NMPA followed by 0.5% LMPA). In the studies in which three layers were used, the first layer consisted of 1% NMPA, while the second and third layers consisted of 0.7% LMPA (Ginzkey et al., 2012). In the studies conducted by Tisch et al. (Gosepath et al., 2003; Hölzer et al., 2008), the first layer consisted of 1% NMPA, the second and third layers consisted of 0.5% LMPA, and the cells were embedded in the third layer. All of the studies that were performed using MOCs utilized only two agarose layers; in nearly all of these studies, the first layer consisted of 0.5% NMPA and the second layer consisted of 0.7% LMPA (Kleinsasser et al., 2004; Sassen et al., 2005; Buehrlen et al., 2007; Baumeister et al., 2009a,b; Reiter et al., 2009; Koehler et al., 2010); differences in the agarose concentration and the composition of the first (1.5% NMPA) and second (0.5% LMPA) layers were applied by Koehler et al. (Hackenberg et al., 2010) 85]. The studies conducted by Hackenberg (Hackenberg et al., 2011; Koehler et al., 2013) only mention that the cells were embedded in LMPA (Table 5).

**Table 5:** **Variations in comet assay protocols employing human nasal epithelial cells**.

**Biomatrix**	**Lysis solution/Duration**	**Enzyme digestion**	**Electrophoresis buffer**	**Unwinding/Electrophoresis duration and V/cm**	**Neutralization buffer**	**Staining**	**Visualization and analysis of images**	**Number of comets**	**Measurement**	**References**
Nasal	2.5 M NaCl, 100 mM Na_2_EDTA, 10 mM Tris pH 10, 1% triton X-100/1 h		1 mM Na_2_EDTA, 300 mM NaOH, pH 13	20 min/20 min, 25 V	0.4 M Tris, pH 7.5	Ethidium bromide		100 comets	Tail length Damage categories	Calderon-Garcidueñas et al., 1996
Nasal	2.5 M NaCl, 100 mM Na_2_EDTA, 10 mM Tris HCL, 1% Na sarcosinate pH 10, 1% triton X-100, 10% DMSO/24 h		1 mM Na_2_EDTA, 300 mM NaOH, pH 13	20 min/20 min, 25 V/300 mA	0.4 M Tris, pH 7.5	Ethidium bromide	Scaled ocular	100 comets	Tail length (microns)	Valverde et al., 1997
Nasal	2.5 M NaCl, 100 mM Na_2_EDTA, 10 mM Tris pH 10, 1% triton X-100/1 h		1 mM Na_2_EDTA, 300 mM NaOH, pH 13	20 min/20 min, 25 V 300 mA	0.4 M Tris, pH 7.5	Ethidium bromide	Scaled ocular	100 comets	Tail length Damage categories	Calderon-Garcidueñas et al., 1999
Nasal	pH 10	-	pH 13	-	-	Ethidium bromide	Scaled ocular	25 representative microscope fields	Tail length	Glück and Gebbers, 2000
Nasal	2.5 M NaCl, 100 mM Na_2_EDTA, 10 mM Tris pH 10, 1% triton X-100, 10% DMSO/1 h	-	1 mM Na_2_EDTA, 300 mM NaOH	20 min/20 min, 0.8 V/cm	0.4 M Tris, pH 7.5	Ethidium bromide	Custom-made imaging software	100 comets	% Tail DNA	Pacini et al., 2003
Nasal	2.5 M NaCl, 100 mM Na_2_EDTA, 10 mM Tris, 1% Na sarcosinate pH 10 1 h		1 mM Na_2_EDTA, 30 mM NaOH, pH 13	20 min/20 min, 25 V/300 mA, 0.8 V/cm	0.4 M Tris, pH 7.5	Ethidium bromide	Scaled ocular	50 comets	Tail length	Fortoul et al., 2003a
Nasal	2.5 M NaCl, 100 mM Na_2_EDTA, 10 mM Tris pH 10 1 h		1 mM Na_2_EDTA, 30 mM NaOH, pH 13	20 min/20 min, 25 V/300 mA, 0.8 V/cm	0.4 M Tris, pH 7.5	Ethidium bromide	Scaled ocular	50 comets	Tail length	Fortoul et al., 2003b
Nasal	2.5 M NaCl, 100 mM Na_2_EDTA, 10 mM Tris pH 10 1 h		1 mM Na_2_EDTA, 30 mM NaOH, pH 13	20 min/20 min, 25 V/300 mA, 0.8 V/cm	0.4 M Tris, pH 7.5	Ethidium bromide	Scaled ocular	50 comets	Tail length	Fortoul et al., 2004
Nasal	2.5 M NaCl, 100 mM Na_2_EDTA, 10 mM Tris pH 10, 1% triton X-100/1 h	UVDE enzyme 1.30 h	1 mM EDTA, 300 mM NaOH	20 min/20 min, 25 V/300 mA	0.4 M Tris - HCl, pH 7.4	Ethidium bromide	Komet 5.0 Kinetic Imaging	100 comets	% Tail DNA	Koreck et al., 2007
Nasal	2.5 M NaCl, 100 mM Na_2_EDTA, 10 mM Tris pH 10 1 h		1 mM Na_2_EDTA, 300 mM NaOH, pH 13	20 min/20 min, 25 V/300 mA	0.4 M Tris, pH 7.5	Ethidium bromide	Scaled ocular	100 comets	Tail length	Fortoul et al., 2010
Nasal epithelial biopsies	2.5 M NaCl, 100 mM Na_2_EDTA, 10 mM Tris HCL, 1% Na sarcosinate pH 10, 1% triton X-100/1–24 h	50 mg protease, 10 mg hyaluronidase, 10 mg collagenase in Juklick medium	1 mM Na_2_EDTA, 300 mM NaOH	20 min/20 min, 25 V/300 mA	0.4 M Tris, pH 7.5	Ethidium bromide	Kinetic Imaging	153 per condition/3 slides	Tail length	Tisch et al., 2002
Nasal epithelial biopsies	-/45 min	Trypsinated in pronase	_	45 min/10 min, 1 V/cm, 300 mA	-	SYBR green	Adobe photoshop	100 comets	Images Tail length Tail width	Gosepath et al., 2003
Nasal epithelium biopsies Nasal epithelial biopsies	2.5 M NaCl, 100 mM Na_2_EDTA, 10 mM Tris HCL, 1% Na sarcosinate pH 10, 1% triton X-100/1–24 h	Proteolytic enzyme	1 mM Na_2_EDTA, 300 mM NaOH	20 min/20 min, 25 V 300 mA	0.4 M Tris, pH 7.5	Ethidium bromide	Kinetic Imaging	51 comets	Tail length	Tisch et al., 2005
Nasal epithelial biopsies	2.5 M NaCl, 100 mM Na_2_EDTA, 10 mM Tris HCL, 1% Na sarcosinate pH 10, 1% triton X-100/1 h	50 mg protease, 10 mg hyaluronidase, 10 mg collagenase in 10 ml Ham's F12	1 mM EDTA, 300 mM NaOH	60 min/20 min, 0.8 V/cm	0.4 M Tris - HCl, pH 7.4	Ethidium bromide	Komet 3.0 Kinetic Imaging	100 comets	Olive tail moment	Hölzer et al., 2008
Nasal epithelial biopsies	2.5 M NaCl, 100 mM Na_2_EDTA, 10 mM Tris pH 10, 1% triton X-100, 10% DMSO/1.5 h	100 μl of enzyme mix containing 0.1 g protease, 1 mg DNAse dissolved in 10 ml phosphate buffered saline were prepared in 9 ml Airway epithelial growth medium	200 mM Na_2_EDTA, 5 mM NaOH	20 min/20 min, 25 V/300 mA	0.4 M Trizma base, pH 7.5	Ethidium bromide	Komet 5.5 Kinetic Imaging	100 comets	Olive tail moment Tail length Tail DNA	Ginzkey et al., 2012
3D Mini organ- cultures of human inferior turbínate epithelia	2.5 M NaCl, 100 mM Na_2_EDTA, 10 mM Tris pH 10, 1% triton X-100, 10% DMSO/1 h	Collagenase P (1 mg/ml), hyaluronidase from bovine testes (1 mg/ml), pronase E type XIV from Streptomyces griseus (5 mg/ml) dissolved in Bronchial Epithelal Basal Medium	1 mM Na_2_EDTA, 300 mM NaOH	20 min/20 min, 25 V/300 mA	0.4 M Tris pH 7.5	Ethidium bromide	Komet 3.1 Kinetic Imaging	80 comets	Olive Tail moment	Kleinsasser et al., 2001
3D Mini organ- cultures of human inferior turbínate epithelia	2.5 M NaCl, 100 mM Na_2_EDTA, 10 mM Tris pH 10, 1% triton X-100, 10% DMSO/1 h	Collagenase P (1 mg/ml), hyaluronidase from bovine testes (1 mg/ml), pronase E type XIV from Streptomyces griseus (5 mg/ml) dissolved in Bronchial Epithelal Basal Medium	1 mM Na_2_EDTA, 300 mM NaOH	20 min/20 min, 25 V/300 mA	0.4 M Tris pH 7.5	Ethidium bromide	Komet 3.1 Kinetic Imaging	80 comets	Olive Tail moment	Kleinsasser et al., 2004
3D Mini organ- cultures of human inferior turbínate epithelia	2.5 M NaCl, 100 mM Na_2_EDTA, 10 mM Tris pH 10, 1% triton X-100, 10% DMSO/90 min	Collagenase P (1 mg/ml), hyaluronidase from bovine testes (1 mg/ml), pronase E type XIV from Streptomyces griseus (5 mg/ml) dissolved in Airway Epithelial Cell Growth Medium	200 mM Na_2_EDTA, 10 mM NaOH	20 min/20 min, 25 V/300 mA	0.4 M Tris, pH 7.5	Ethidium bromide	Komet 4.0 Kinetic Imaging	80 comets	Olive Tail moment	Sassen et al., 2005
3D Mini organ- cultures of human inferior turbínate epithelia	2.5 M NaCl, 100 mM Na_2_EDTA, 10 mM Trizma Base, 1% Na sarcosinate pH 10, 1% triton X-100, 10% DMSO/ 90 min	Collagenase P (1 mg/ml), hyaluronidase (1 mg/ml), protease (5 mg/ml) dissolved in Bronchial Epithelal Basal Medium	1 mM Na_2_EDTA, 300 mM NaOH	20 min/20 min, 25 V/300 mA, 1 V/cm	0.4 M Trizma Base, pH 7.5	Ethidium bromide	Komet 3.1 Kinetic Imaging	80 comets	Olive Tail moment	Buehrlen et al., 2007
3D Mini organ- cultures of human inferior turbínate epithelia	DMSO 10 ml, 1 ml Triton X-100, 89 ml alkaline lysis (NaCl 0.9%, Na_2_EDTA, Trizma base, N- laurylsarcosin sodium salt)/1 h	Collagenase P (1 mg/ml), hyaluronidase from bovine testes (1 mg/ml), pronase E type XIV from Streptomyces griseus (5 mg/ml) dissolved in Bronchial Epithelal Basal Medium	200 mM Na_2_EDTA, 10 mM NaOH	20 min/20 min, 300 mA, 0.8 V/cm	0.4 M Trizma Base, pH 7.5	Ethidium bromide	Kinetic Imaging	40 comets	Olive Tail moment	Reiter et al., 2009
3D Mini organ- cultures of human inferior turbínate epithelia	2.5 M NaCl, 100 mM Na_2_EDTA, 10 mM Trizma Base, 1% Na sarcosinate pH 10, 1% triton X-100, 10% DMSO	Collagenase P (1 mg/ml), hyaluronidase (1 mg/ml), protease (5 mg/ml) FPG	1 mM Na_2_EDTA, 300 mM NaOH	20 min/20 min, 25 V/300 mA, 1 V/cm	0.4 M Trizma Base, pH 7.5	Ethidium bromide	Komet Kinetic Imaging	80 comets	Olive tail moment	Baumeister et al., 2009a
3D Mini organ- cultures of human inferior turbínate epithelia		Collagenase P (1 mg/ml), hyaluronidase (1 mg/ml), protease (5 mg/ml) FPG	-	-	-	Ethidium bromide	Komet 3.1 Kinetic Imaging	80 comets	Olive tail moment	Baumeister et al., 2009b
3D Mini organ- cultures of human inferior turbínate epithelia	2.5 M NaCl, 100 mM Na_2_EDTA, 10 mM Tris pH 10, 1% triton X-100, 10% DMSO/1.5 h	Collagenase P (1 mg/ml), hyaluronidase (1 mg/ml), protease (5 mg/ml) FPG	200 mM Na_2_EDTA, 5 mM NaOH	20 min/20 min, 25 V/300 mA	0.4 M Trizma Base, pH 7.5	Ethidium bromide	Komet 4.0 Kinetic Imaging	80 comets	Olive tail moment Tail length	Koehler et al., 2010
3D Mini organ- cultures of human inferior turbínate epithelia	2.5 M NaCl, 100 mM Na_2_EDTA, 10 mM Tris pH 10, 1% Triton X-100, 10% DMSO/1.5 h	9 ml Minimum Essential Medium, 0.1 mg/ml protease XIV, 1 mg/ml DNAse DN25, 0.05 mg/ml gentamycin, 100 U/ml penicillin with 1 μg/ml streptomycin, 0.250 U/ml amphotericin B and 2 mM L- glutamine	200 mM Na_2_EDTA, 5 mM NaOH	20 min/20 min, 25 V/300 mA	0.4 M Tris, pH 7.5	Ethidium bromide	Komet 5.0 Kinetic Imaging	100 comets	Olive tail moment Tail length Tail DNA	Hackenberg et al., 2010
3D Mini organ- cultures of human inferior turbínate epithelia	2.5 M NaCl, 100 mM Na_2_EDTA, 10 mM Tris pH 10, 1% Triton X-100, 10% DMSO/1.5 h	9 ml Minimum Essential Medium, 0.1 mg/ml protease XIV, 1 mg/ml DNAse DN25, 0.05 mg/ml gentamycin, 100 U/ml penicillin with 1 μg/ml streptomycin, 0.250 U/ml amphotericin B and 2 mM L- glutamine	200 mM Na_2_EDTA, 5 mM NaOH	20 min/20 min, 25 V/300 mA	0.4 M Tris pH 7.5	Ethidium bromide	Komet 5.5 Kinetic Imaging	100 comets	Olive tail moment Tail length Tail DNA	Hackenberg et al., 2011
3D Mini organ- cultures of human inferior turbínate epithelia	2.5 M NaCl, 100 mM Na_2_EDTA, 10 mM Tris pH 10, 1% Triton X-100, 10% DMSO/1.5 h	Collagenase P (1 mg/ml), hyaluronidase (1 mg/ml), protease (5 mg/ml)	200 mM Na_2_EDTA, 5 mM NaOH	20 min/20 min, 25 V/300 mA	0.4 M Trizma base, pH 7.5	Ethidium bromide	Komet 5.5 Kinetic Imaging	100 comets	Olive tail moment Tail length Tail DNA	Koehler et al., 2013

The remainder of the comet assays that were performed in nasal cells all followed the alkaline version of the protocol that was proposed by Singh et al. (1988), with very few modifications. After generating the slides, the cells were exposed to a lysis solution (1% sodium sarcosinate, 2.5 M NaCl, 100 mM Na_2_ EDTA, and 10 mM Tris-base, pH 10, containing 10% DMSO, and 1% Triton X-100) for a minimum of 1 h; however, certain studies did not include the addition of sodium sarcosinate, DMSO or Triton X-100. Enzyme addition to detect specific DNA damage was only performed in two of the studies; Koreck et al. (Fortoul et al., 2010) used the enzyme UVDE to detect cyclobutane pyrimidine dimers, and Baumeister et al. (Koehler et al., 2010) used formamidopyrimidine glycosylase, which specifically recognizes 8-hydroxy-guanines (Table 5).

To allow the DNA to unwind, an alkaline electrophoresis buffer was used; during this step, 70% of the studies used 1 mM Na_2_ EDTA and 300 mM NaOH (pH > 13), while the remaining 30% employed a buffer containing 200 mM Na_2_ EDTA and 5–10 mM NaOH (Kleinsasser et al., 2001; Buehrlen et al., 2007; Baumeister et al., 2009a; Hackenberg et al., 2010, 2011; Koehler et al., 2013). With respect to the duration of unwinding and electrophoresis, 90% of the studies utilized 20 min for unwinding and 20 min for electrophoresis, applying a current of 25 V and 300 mA (ranging from 0.8 to 1 V/cm). Following electrophoresis, the alkaline conditions were neutralized using a 0.4 M Tris (pH 7.5) solution. The slides were subsequently stained with ethidium bromide, with the exception of one study, in which SYBR Green was used (Gosepath et al., 2003) (Table 5).

Evaluation of the slides was performed using either scaled ocular or specialized software to measure tail length (45%), % tail DNA (11%) and Olive tail moment (41%), with the exception of one study, which measured only the tail length and width (3%) (Tisch et al., 2005). The number of comets evaluated per slide ranged from 50 to 153 (Table 5).

### Results

As mentioned previously, comet assay studies utilizing human nasal epithelial cells may be divided into three groups based on the sampling procedure that was used: Cytobrush-obtained, biopsies and MOCs. Methodologically, the nasal cell studies using direct sampling from subjects approached the comet assay in similar manners (Valverde et al., 1997; Calderon-Garcidueñas et al., 1999; Glück and Gebbers, 2000; Tisch et al., 2002; Fortoul et al., 2003a,b, 2004, 2010; Pacini et al., 2003; Koreck et al., 2007). The majority of the studies that were used to determine air pollution-induced DNA damage obtained positive results using the comet assay; specifically, certain studies also revealed a correlation between DNA damage and ozone exposure (Valverde et al., 1997; Calderon-Garcidueñas et al., 1999; Glück and Gebbers, 2000; Tisch et al., 2002; Fortoul et al., 2003a; Koreck et al., 2007). Two of the studies that were conducted by Fortoul et al. (2003b, 2004) detected increased DNA damage in nasal epithelial cells from asthmatic individuals. Meanwhile, the study conducted by Koreck et al. (Fortoul et al., 2010) determined that more DNA damage was induced by phototherapy and utilized a repair assay to determine that the induced damage was removed after 10 days; it is also important to mention that this study was the only study to utilize enzymatic digestion to analyze specific DNA lesions.

Five studies utilized nasal cavity biopsies, and all of these studies established primary cultures that were treated *ex-vivo*. Gosepath et al. (Tisch et al., 2005) did not report the use of enzymatic digestion of the specimens to obtain the cell suspensions that were used in the comet assay. This study reported the induction of DNA damage following benzene treatment for a period of 8 h, and this damage persisted after 24 h. Tisch et al. (Gosepath et al., 2003; Hölzer et al., 2008) examined the genotoxicity of pesticides and found that permethrin, DEET, diazinon, pentachlorophenol, lindane, transfluthrin, cyfluthrin and pyrethrum induced DNA damage in nasal epithelial cells; these authors performed enzymatic digestion prior to conducting the comet assay. Hölzer et al. (Ginzkey et al., 2012)compared the genotoxic potential of various chemicals (N-nitrosodiethanolamine, epichlorohydrin, 1,2-epoxibutane, ethylene dibromide, and 1,2-dibromo-3-chloropropane)in nasal epithelial cells that had been derived from rats and humans, and found that human cells were less sensitive than rat mucosal cells to the genotoxic activities of N-nitrosodiethanolamine, ethylene dibromide, and 1,2-dibromo-3-chloropropane, while similar levels of DNA damage induction were observed for epichlorohydrin and 1,2-epoxybutane. Ginzkey et al. (Kleinsasser et al., 2001) found that nicotine increased DNA damage, which was prevented by NAC and mecamylamine.

The comet assay results for *ex-vivo* MOC exposure were primarily generated by the Kleinsasser group, which used the assay to determine chemically induced DNA damage (Kleinsasser et al., 2004; Sassen et al., 2005; Buehrlen et al., 2007; Reiter et al., 2009; Hackenberg et al., 2010, 2011; Koehler et al., 2013). The chemicals tested included: N-nitrosodiethylamine, sodium dichromate, N-methyl-N-nitro-N-nitrosoguanidine, mono (2-ethylhexyl) phthalate, benzo[a]pyrene-7,8-diol-9,10-epoxyde, nicotine, nitrogen dioxide, zinc oxide, and titanium dioxide nanoparticles. DNA damage was induced by all of these chemicals, with the exception of titanium dioxide nanoparticles. The Harreus group also examined MOCs, in which they studied the chemopreventive activity of several compound following oxidative challenge with H_2_O_2_ or dexamethasone (Baumeister et al., 2009a,b; Koehler et al., 2010); these authors found that NAC, α-tocopherol, quercetin, coenzyme Q10, ascorbic acid, and zinc reduced DNA damage in nasal epithelial cells.

### Discussion

Application of the comet assay to determine genotoxicity in nasal epithelial cells does not require modifications to the cell lysis and electrophoresis steps of the protocol that was outlined by Singh et al. (1988). The most important point in the use of nasal epithelial cells is the sampling method. A consensus in the sampling methods used in the three previously mentioned variations was reached. First, cells obtained using a cytobrush are only required to be maintained in cold medium and processed as soon as possible. Second, the most important observation is that the procedure can be considered to be a non-invasive procedure. Biopsies that were used to establish primary cultures required enzymatic digestion before they could be used in the comet assay. Until now, these types of studies have been published periodically; however, only five studies have determined that the nasal epithelium serves as a superior biomatrix to other cell types when assessing DNA damage that is induced by inhaled chemicals. In addition, a consensus was achieved in the sampling procedure when working with 3D miniorgan-cultures, because all of the studies reported the use of enzymatic digestion via solutions with similar compositions by altering the physiological solution to dissolve the enzymes (Kleinsasser et al., 2004; Sassen et al., 2005; Buehrlen et al., 2007; Baumeister et al., 2009a,b; Reiter et al., 2009; Hackenberg et al., 2010, 2011; Koehler et al., 2010, 2013). The congruence in the procedure resulted from the fact that only two groups applied this biomatrix: The Kleinsasser (Kleinsasser et al., 2004; Sassen et al., 2005; Buehrlen et al., 2007; Reiter et al., 2009; Hackenberg et al., 2010, 2011; Koehler et al., 2013) and Harreus groups (Baumeister et al., 2009a,b; Koehler et al., 2010). The homogeneity between the procedures validated the assay because comparison of basal DNA damage reflected low variability; therefore, it may be important for new studies to employ this cell type and apply the procedure that was previously established by these groups.

## Guidelines

It is obvious that generate a unified protocol for all kind of epithelial cells is impossible. However, this section provides a general comet assay procedure for *ex-vivo* and *in-vivo* epithelium samples.

In the present paper, we mention the advantages and shortcomings of the use of alternative biomatrices to assess DNA damage in human populations, focusing on the methodological characteristics of each type of epithelium and taking the sampling protocol, pre-processing, and post-sampling storage into consideration, as well as the possibilities of sample (snap) freezing and the need to adapt the classical alkaline comet protocol. The advantages to use epithelial cells to mapping DNA damage by comet assay is the possibility to obtain samples with non-invasive methodologies for *in-vivo* studies in a safety and cheapest way. Epitheliums are in direct contact with xenobiotics and endogenous damage inductors, being an attractive biomatrice to evaluate individual genotoxicity to several compounds in the case of 3D miniorgans establish by nasal epithelium. Their applicability in clinical diagnostic confers a potential use in patients across time. Some disadvantages to take in account are the invasive procedures for *ex-vivo* studies, the expensive cost to sampling just to determine DNA damage; however is a perfect possibility to realize multidisciplinary studies when the invasive procedure is required.

The general guideline to realize comet assay in epithelial cells require the correct sampling procedure, to follow the alkaline version proposed by Singh et al. (1988). Sampling differ between *ex-vivo* and *in-vivo* procedures, in this sense we porpoise protocols to specific epithelium source in early sections (Sections Sampling Protocol and Sample Storage, Comet Assay Sample Preparation for lens; Sampling Protocol and Sample Storage, Comet Assay Sample Preparation for corneal; Sampling Protocol and Sample Storage, Comet Assay Sample Preparation for tear duct; Sampling Protocol and Sample Storage, Comet Assay Sample Preparation for buccal; Sampling Protocol and Sample Storage, Comet Assay Sample Preparation for nasal cells).

### Lens epithelial cells

Slide preparation. Pre-coated with 0.75% NMPA as fist layer, second layer with 1% LMPA mixed with cell suspension.

Lysis solution. The original recipe (2.5 M NaCl, 100 mM Na_2_ EDTA, 10 mM Tris HCL, 1% Na sarcosinate pH 10, in fresh add 1% triton X-100, 10% DMSO) during overnight incubation.

Electrophoresis solution. The original recipe (1 mM Na_2_ EDTA, 300 mM NaOH, pH > 13).

Unwinding and electrophoresis. Incubation periods of 20 min, close to 300 mA.

Neutralization solution. The original recipe (0.4 M Tris pH 7.5^*^ for EtBr stain^*^).

Number of nucleoids evaluated. 50 comets.

### Corneal cells

Slide preparation. Pre-coated with 1% NMPA as fist layer, second layer with 1% LMPA mixed with cell suspension.

Lysis solution. The original recipe (2.5 M NaCl, 100 mM Na_2_ EDTA, 10 mM Tris HCL, 1% Na sarcosinate pH 10, in fresh add 1% triton X-100, 10% DMSO) during overnight incubation.

Electrophoresis solution. The original recipe (1 mM Na_2_ EDTA, 300 mM NaOH, pH > 13).

Unwinding and electrophoresis. Incubation periods of 20 min, close to 300 mA.

Neutralization solution. PBS, H_2_O^**^ (For SYBR stain^**^).

Number of nucleoids evaluated. 50 comets.

### Tear duct cells

Slide preparation. Pre-coated with 0.5% NMPA as fist layer, second layer with 0.5% LMPA mixed with cell suspension, and third layer with 0.5% LMPA.

Lysis solution. The original recipe (2.5 M NaCl, 100 mM Na_2_ EDTA, 10 mM Tris, pH 10, in fresh add 1% triton X-100, 10% DMSO) during 1 h incubation.

Electrophoresis solution. The original recipe (1 mM Na_2_ EDTA, 300 mM NaOH, pH > 13).

Unwinding and electrophoresis. Incubation periods of 20 min, close to 300 mA.

Neutralization solution. The original recipe (0.4 M Tris pH 7.5^*^ for EtBr stain^*^).

Number of nucleoids evaluated. 50 comets.

### Buccal epithelial cells

Slide preparation. Pre-coated with 0.5% NMPA as fist layer, second layer with 0.5% LMPA mixed with cell suspension, and third layer with 0.5% LMPA.

Lysis solution 1. 2.5 M NaCl, 100 mM Na_4_ EDTA, 10 mM Tris HCL, 1% Na sarcosinate pH 10, in fresh add 1% triton X-100, 10% DMSO, during 1 h incubation.

Lysis solution 2. Fresh solution (2.5 M NaCl, 100 mM Na_4_ EDTA, 10 mM Tris HCL, 1% Na sarcosinate pH 10, in fresh add 1% triton X-100, 10% DMSO) add Proteinase K (1 mg/ml) warm to 37°C and 1 h incubation.

Electrophoresis solution. The original recipe (1 mM Na_2_ EDTA, 300 mM NaOH, pH > 13).

Unwinding and electrophoresis. Incubation periods of 20 min, close to 300 mA.

Neutralization solution. The original recipe (0.4 M Tris pH 7.5^*^ for EtBr stain^*^).

Number of nucleoids evaluated. 50 comets.

### Nasal cells

Slide preparation. Pre-coated with 0.5% NMPA as fist layer, second layer with 0.5% LMPA mixed with cell suspension, and third layer with 0.5% LMPA.

Lysis solution. The original recipe (2.5 M NaCl, 100 mM Na_2_ EDTA, 10 mM Tris, pH 10, in fresh add 1% triton X-100, 10% DMSO) during 1 h incubation.

Electrophoresis solution. The original recipe (1 mM Na_2_ EDTA, 300 mM NaOH, pH > 13).

Unwinding and electrophoresis. Incubation periods of 20 min, close to 300 mA.

Neutralization solution. The original recipe (0.4 M Tris pH 7.5^*^ for EtBr stain^*^).

Number of nucleoids evaluated. 50 comets.

Our suggestion is follow the comet assay procedure in a close way to the Singh et al. (Singh et al., 1988) protocol to diminish the variability between groups. In addition, is important consider that buccal epithelial cells is the unique cell type that require lysis enrichment with proteinase K to obtain free nucleosomes as part of the comet assay protocol.

There are different modifications that needs to be imporved during sampling to obtain a cellular suspension friendly to comet assay or primary culture stablishment.

## Conclusions

Over a 30 year period, the comet assay has been employed in molecular epidemiology as a robust biomarker of the early effects of diseases on human populations. Over the past 10 years in particular, the alkaline assay has been shown to play an important role in monitoring the effects of occupational and environmental hazards. The applicability of the comet assay to almost any cell type confers the important advantage of exploring the use of other biomatrices, such as epithelial cells.

Epithelia are sheets of cells that either line the walls of cavities and channels or, in the case of skin, serve as the outside covering of the body. By the first decades of 20th century, detailed histological analyses had revealed that normal tissues containing epithelia are all structured similarly (Kruze, 1994). In addition, the possibility of obtaining epithelial cells using biopsies or less invasive procedures was the perfect match for applying the comet assay to evaluate DNA damage.

The studies reviewed in the present manuscript can be clearly divided into one of two groups: Those with clear clinical applications (lens and corneal epithelial cells) and those examining the use of epithelial cells as biomarkers for genotoxicity assessments in human monitoring and under *in vitro* conditions.

In the first group, lens cells have been shown to be a useful tool for DNA damage detection in individuals with cataracts. This pathology primarily results from oxidative stress and UV radiation, with these cells producing opacity and developing genotoxicity that can be detected using the alkaline comet assay. These factors suggest that the comet assay may be applied to understand other eye pathologies, such as macular degeneration. Corneal cells also fall in to this group and have been used with the aim of determining DNA damage in cells with the potential to be transplanted, although additional damage may be induced by the manipulation. With respect to both of these cell types, few studies have been conducted (Tables 1, 2). However, the studies that have been conducted suggest the feasibility of their use in toxicology, pharmacy, regenerative medicine, and tissue culture.

The group in which epithelial cells were used as genotoxicity biomarkers in human monitoring involves studies using tear duct, buccal, and nasal epithelial cells (Tables 3–5). A tear duct study determined genotoxicity in humans that had been exposed to air pollution, which generated ophthalmological symptomatology. Therefore, exfoliated eye cells may be a sensitive target for the genotoxic evaluation of ophthalmological products, cosmetics, and gasses that may come in direct contact with the eye. The use of buccal epithelial cells in the comet assay has versatility in determining genotoxicity, such as the use of the micronucleus test. Over the past few years, the use of these types of cells in human monitoring has increased in the field of odontology, evaluating several types of chemicals, and odontological procedures, because buccal epithelial cell renewal occurs every 10–14 days. The nasal epithelial cell renewal rate is approximately once every 30 days, reflecting their utility in detecting DNA damage that is induced by the interaction of several substances or environmental conditions during a recent period of exposure when the comet assay is applied during human monitoring. However, over the past 10 years, the use of nasal epithelial cells has been proposed to establish 3D cultures of these cells and determine genotoxicity using an *in vitro* model.

At present, epithelial cells are not sufficiently utilized for genotoxicity evaluations. An important argument for using epithelial cells is that the majority of human tumors arise from epithelial tissues. Detection of DNA damage in this cell types of cells can be done on single level using the comet assay.

### Conflict of interest statement

The authors declare that the research was conducted in the absence of any commercial or financial relationships that could be construed as a potential conflict of interest.
